# Carbon Materials in Electroanalysis of Preservatives: A Review

**DOI:** 10.3390/ma14247630

**Published:** 2021-12-11

**Authors:** Slawomir Michalkiewicz, Agata Skorupa, Magdalena Jakubczyk

**Affiliations:** Institute of Chemistry, Jan Kochanowski University, PL-25406 Kielce, Poland; agata.skorupa@ujk.edu.pl (A.S.); magdalena.jakubczyk@ujk.edu.pl (M.J.)

**Keywords:** carbon materials, electrode, voltammetry, preservatives, determination

## Abstract

Electrochemical sensors in electroanalysis are a particularly useful and relatively simple way to identify electroactive substances. Among the materials used to design sensors, there is a growing interest in different types of carbon. This is mainly due to its non-toxic properties, low cost, good electrical conductivity, wide potential range, and the possibility of using it in both aqueous and nonaqueous media. The electrodes made of carbon, and especially of carbon modified with different materials, are currently most often used in the voltammetric analysis of various compounds, including preservatives. The objective of this paper is to present the characteristics and suitability of different carbon materials for the construction of working electrodes used in the voltammetric analysis. Various carbon materials were considered and briefly discussed. Their analytical application was presented on the example of the preservatives commonly used in food, cosmetic, and pharmaceutical preparations. It was shown that for the electroanalysis of preservatives, mainly carbon electrodes modified with various modifiers are used. These modifications ensure appropriate selectivity, high sensitivity, low limits of detection and quantification, as well as a wide linearity range of voltammetric methods of their identification and determination.

## 1. Introduction

A characteristic feature of the modern world is an increasing demand for products with a long shelf-life. This mainly applies to the products of the food, pharmaceutical, and cosmetic industries. Maintaining appropriate quality, microbiological purity, durability, and proper organoleptic characteristics of a product is possible thanks to the use of preservatives. Preservatives are a wide range of substances that, when added to a pharmaceutical drug, food, or cosmetic, protect against the growth of microorganisms such as bacteria, fungi, and mold. If the term “preservative” is understood as a substance that is introduced into a product to extend its shelf-life [1,2], then antioxidants should also be included in this group [3]. This is because the durability of preparation also depends on effective protection against oxidation. To achieve this goal, chemical compounds that act as antioxidants are added to products. The most popular preservatives for food, pharmaceuticals, and cosmetics are benzoic acid, parabens, phenoxyethanol, and synthetic antioxidants (BHA and BHT). The adverse impact of these preservatives on human health makes it necessary to develop and improve the methods of their determination in everyday products. For this purpose, mainly chromatographic techniques, rarely spectrophotometric and electrochemical ones, are used.

Electroanalysis is a practical part of electrochemistry designed to solve real-life analytical problems. They are associated mainly with the identification and quantification of elements or chemical compounds. In recent years, there has been a dynamic development of electrochemical methods, especially voltammetric ones. Currently, they have become an attractive alternative to commonly used chromatographic methods. In terms of accuracy, precision, limits of detection, and determination, they are comparable to chromatographic methods but do not require expensive and complex equipment [4,5,6]. Another asset is the simplicity of sample preparation for analysis, which shortens the time of this stage, especially in the case of biological matrices. For the voltammetric analysis of electroactive compounds, derivatization is not necessary. Sample preparation is often limited to its dissolution in a suitable solvent, and optionally, to filtration separating insoluble matrix components. Their use may limit the presence of other electroactive components of a sample, the signals of which may interfere with this of the analyte [7,8,9]. These advantages can shorten the time of analysis and minimize the possibility of errors. Thanks to the miniaturization of the measuring cell, it is also possible to significantly reduce the consumption of reagents, which minimizes the costs of analysis and makes voltammetric techniques eco-friendly [10].

The basis of voltammetric techniques is usually oxidation and/or reduction reactions proceeded on the surface of the working electrode. These heterogeneous reactions provide both qualitative and quantitative information on the analytes. The key to obtaining optimal analytical parameters of the electroanalytical method is the selection of appropriate measurement conditions. This applies to the type of solvent and supporting electrolyte as well as the voltammetric technique. The material of the working electrode is also of great importance. Since redox reactions are associated with electron exchange, the electrode materials for voltammetry must be conductive. The choice is thus limited to metals, conductive solids, or semiconductors. The material used for the construction of the electrode must meet several other important conditions [6,11,12,13]: (i) inert towards the solvent and the supporting electrolyte; (ii) wide potential range, called the potential window, enabling the investigated electrode reaction to proceed; (iii) low background current and high signal-to-noise ratio; (iv) reproducible response; (v) resistance to poisoning and blocking the surface in complex matrices and by the products of the electrode reaction; (vi) easy surface regeneration by cleaning or polishing.

Several materials have been used as working electrodes. Mercury was the first metal that Jaroslav Heyrovsky applied in the form of a dropping electrode (DME), as a working electrode, in polarography [14]. For many years, DME was an essential voltammetric electrode commonly used to investigate reduction reactions of both inorganic and organic compounds. Its popularity was due to many attractive properties, including a highly reproducible, renewable, smooth surface and a wide cathodic potential range. Heyrovsky’s significant contribution to the development of voltammetry was recognized with the award of the Nobel Prize in 1959 [10]. Both DME and other modern electrode designs, such as a hanging mercury drop electrode (HMDE), a controlled growth mercury electrode (CGMDE), or a mercury film electrode (MFE), have been widely used in electroanalysis [11,12,15]. It is well known that there is no electrode material as good as mercury for studying reductions reactions [5]. The disadvantages of the use of mercury are its limited anodic potential range and, in particular, its toxicity. Therefore, alternative, non-toxic green chemistry compliant electrode materials are highly desirable.

Various non-mercury electrode materials were applied in voltammetric studies. Noble metals, especially platinum, gold, silver, and copper, are widely applied solid materials. Rhodium, palladium, germanium, gallium, and lead are used much less frequently. The disadvantage of metal electrodes is their susceptibility to poisoning and blocking the surface with products of electrode reactions, particularly products of oxidation of organic compounds with phenolic structure [16]. The electrodes made of metal oxides or polymers, and those with chemically modified surfaces, are also used. Solid electrodes are of different sizes and geometries. The most common ones are planar disc electrodes in a stationary or rotating working mode. Such electrodes consist of a cylindrical rod of the electrode material embedded in an insulating tube made of Teflon, glass, etc. Recently, one of the most popular electrode materials was environmentally friendly carbon of various forms, as well as those with surface modified with many compounds [12,17].

The present study reviewed various carbon materials used for the construction of working electrodes, as well as their application in the voltammetric analysis of the most popular preservatives.

## 2. Carbon Materials for Working Electrodes in Voltammetry

**Figure 1:**
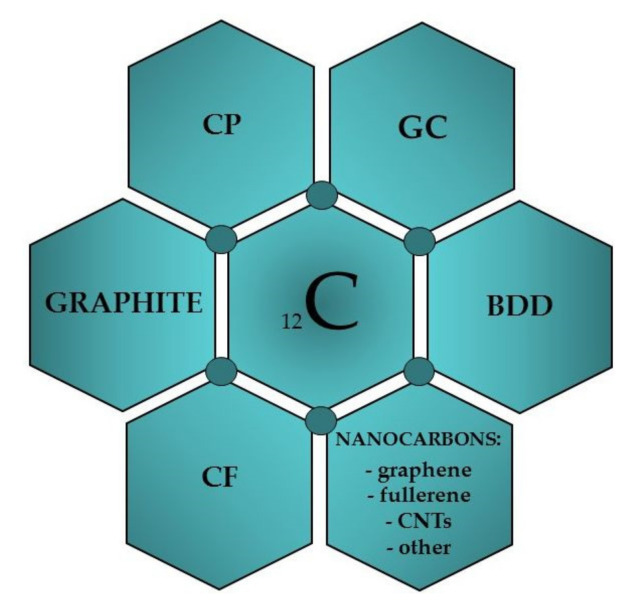
Carbon materials used to construct voltammetric electrodes.

The history of using carbon as an electrode material dates back to the beginning of the 19th century when Humphrey Davy used a graphite electrode for the electrowinning of alkali metals [18,19]. The material in the form of carbon paste (CP) was used for the first time in electroanalysis in 1958 by R.N. Adams [20]. CP was designed as an alternative to a dropping mercury electrode and was used by the author in “*anodic polarography*” for the oxidation of iodide ions. Since then, the use of carbon as a working electrode material has increased rapidly, and it has become widely used in electroanalysis today [11,13,18,19,21,22]. Its popularity is mainly due to nontoxic properties, low cost, availability of various types of carbon materials, good electrical conductivity, wider potential range than obtained on metal electrodes, low background currents, chemical and electrochemical inertness and stability, the possibility of use in both aqueous and nonaqueous media, and easiness of surface modification via strong covalent or noncovalent bonding with surface modifiers, which improves its electrochemical performance [18,23,24]. In contrast to solid metal electrodes, electron-transfer rates observed on carbon surfaces are often slower. Due to the high active surfaces of carbon electrodes, they are susceptible to adsorption of organic compounds and, consequently, to poisoning, which blocks the access of analytes to their surfaces and hinders the electron exchange reaction. Obtaining reproducible results requires a cleaning procedure that depends on the type of carbon material used. Practically, all carbon forms, and their allotropes, were used for the construction of working electrodes in voltammetry (Figure 1): glassy carbon (GC), carbon paste (CP), carbon fiber (CF), boron-doped diamond (BDD), different types of graphite (G), fullerenes (C60), graphene (GN), carbon nanotubes (CNTs), screen-printed carbon (SPC), carbon with the modified surface, etc. [18,21,22,25]. A brief characteristic of the main carbon materials used in the voltammetric analysis is presented below.

### 2.1. Glassy Carbon

Glassy carbon (GC), also called vitreous carbon, is the most common carbon material used to produce voltammetric working electrodes. It started to be manufactured in the early 1960s by carefully controlled heating of a phenol-formaldehyde resin body in an inert atmosphere. The pyrolysis process proceeds very slowly over the 300–1200 °C temperature range [11,12,13,15]. This material contains carbon with sp^2^ hybridization; it is macroscopically isotropic with the same electrical properties in all directions, hard, chemically resistant, and of high density because the existing pores are tightly closed. Thus, no impregnation procedure is required. It consists of cross-linked graphite-like sheets. GC is placed in a fitting tube of an insulating material to construct a stationary or rotating disk glassy carbon electrode (GCE) [11,12]. The surface of the GCE is cleaned by polishing it with alumina oxide and rinsing it with deionized water before use. Sometimes, chemical or electrochemical activation is required. Glassy carbon with a bare and modified surface has been the most often used electrode material since the 1980s [11,13,15,26]. GC surface is often modified, e.g., with a film of carbon nanotubes (CNTs), graphene, chemically reduced graphene oxide, fullerenes [26,27] metal oxides: In_2_O_3_ [28], CuO/Cu_2_O [29], IrO_x_ [30], or their composites with reduced graphene oxide: R-GNO/ZnO [31], MnO_2_/R-GNO [32]. An interesting modifier of the surface of GCE is the hybrids of cobalt oxides nanoparticles with graphene oxide (GNO/Co_3_O_4_) [33], reduced graphene oxide (R-GNO/Co_3_O_4_) [34] or with multi-walled carbon nanotubes (MWCNT/Co_3_O_4_) [35]. Recently, a “hot” material used to modification of GCE surface is a composite of α-zirconium phosphate with graphitic carbon nitride (α-ZrP@G-C_3_N_4_/GCE) used for sensitive detection of nitrite ions (NO_2_^−^) [36]. The composite G-C_3_N_4_/MWCNTs deposited on the GCE surface was recently applied to sensitive detection of Cu(II) ions [37].

### 2.2. Graphite

**Figure 2:**
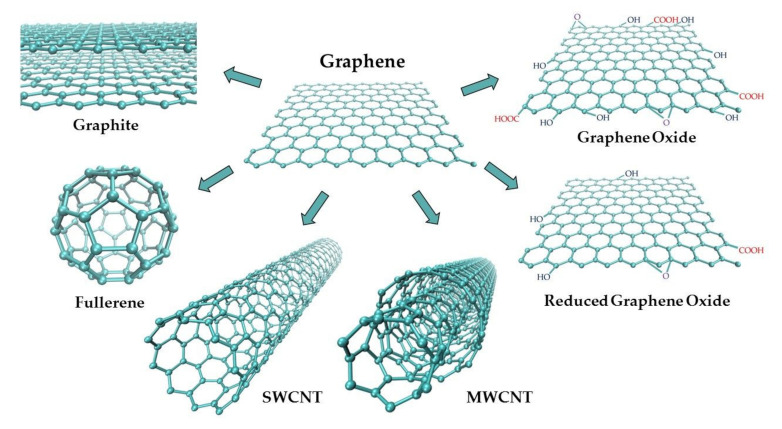
Schematic representation of graphene-based carbon materials.

Graphite (G) is a widespread natural allotrope of carbon with the highest thermodynamic stability [23,24]. Its structure consists of parallel layers with conjugated, hexagonal aromatic carbon rings (sp^2^ hybridization), with both σ and π bonds (Figure 2). The existence of delocalized π orbitals allows for the free movement of electrons parallel to the layers, and therefore, graphite exhibits relatively high electric conductivity. Thus, it is a good material for working electrodes.

**Figure 3:**
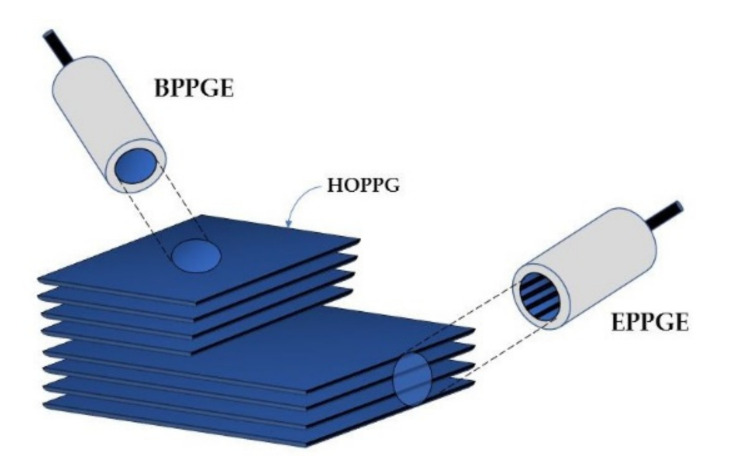
Construction of basal plane and edge plane pyrolytic graphite electrodes.

Graphite layers are connected to each other by weak van der Waals forces, which makes them easy to separate [13,38,39]. Consequently, this softest carbon allotrope is susceptible to abrasion. Synthetic graphite is obtained by the controlled pyrolysis of light hydrocarbons (ca. 800 °C). The product undergoes heat treatment at higher temperatures. Pyrolytic Graphite (PG) is then converted into Highly Ordered Pyrolytic Graphite (HOPG) by annealing at ca. 3000 °C, at a pressure of several kilobars [15]. HOPG (Figure 3) is most often used in the construction of graphite working electrodes [18]. The anisotropic structure of HOPG causes that, depending on its cutting mode, two electrodes of different properties can be obtained: Edge Plane Pyrolytic Graphite Electrode (EPPGE) and Basal Plane Pyrolytic Graphite Electrode (BPPGE) [26,40]. As a result of the cutting of HOPG along its crystal lattice, BPPGE is obtained. EPPGE, on the other hand, is formed by cutting across the crystal lattice (Figure 3). These electrodes differ in their electrochemical properties. The perpendicular arrangement of graphite layers in EPPGE causes that it exhibits much higher conductivity compared to BPPGE, and it is more reactive towards electron transfer [13,40]. It was found that the electrode kinetics at EPPGE is at least three times faster than at BPPGE [26,40]. EPPGE is also characterized by strong adsorption properties, a wide potential window, relatively low background currents, as well as mechanical and corrosion resistance.

An interesting electrode material is pencil graphite (PG). It is produced in an aqueous environment, from a mixture of graphite powder and clay as a binder material, then heated up to 1000 °C in order to gain rigidity. The use of wax allows pores to be filled and gives pencil graphite a smooth appearance [41]. The use of modified pencil graphite electrodes (PGEs), as biosensors comprising enzymes, nucleic acids, and other biological entities, for the determination of organic compounds (e.g., glucose, ascorbic acid, cholesterol, uric acid) was recently reviewed by Torrinha et al. [41].

### 2.3. Diamond

Next to graphite, diamond is the second known natural allotrope of carbon. It has a cubic, close-packed structure with sp^3^ hybridization of each carbon atom. This mineral is transparent, brilliant, and is the hardest known substance in nature [13,18]. Apart from this, it is extraordinary chemically stable and has a very high melting point. Unlike other forms of carbon, diamond is a very poor electrical conductor and is useless as an electrode material [42]. Its conductivity increases after the intentional introduction of impurities, most often of boron [18,42]. Boron-doped diamond (BDD) thin film is made by means of chemical vapor deposition from hydrogen plasma containing methane and source of boron, often trimethylboron, trimetyl borane, or B_2_H_6_ [18,43]. Boron doping level is usually high, and the B/C ratio reaches 10^−5^ to 10^−3^ [18]. A boron-doped diamond electrode (BDDE) has electrochemical properties compatible with graphitic materials. However, it is much more chemically inert, as well as corrosion and mechanically resistant. It also exhibits negligible susceptibility to adsorption of organic compounds. BDDE has a very low and stable background current and a very wide potential range, both in an aqueous and organic medium (3–3.5 V and 5–7.5 V, respectively) [17,18,23,42,43,44,45]. These unique properties have caused BDDE to become an important tool in electrochemistry since the early 1990s [46,47]. The electrochemical properties of this electrode strongly depend on its surface termination [17,44,45,48,49]. The activation process is usually carried out in an acidic medium of sulfuric acid. The cathodic pre-treatment at a high negative potential (in the hydrogen evolution region) causes a reduction in the surface, and thus it is terminated with hydrogen (H-termination). The anodic pre-treatment takes place at a high positive potential (in the water decomposition region) and causes oxidation of the surface and its termination with oxygen (O-termination). H-termination of the BDD makes its surface more hydrophobic than in the case of O-termination [42,44,45]. These differences have a crucial influence on electron transfer kinetics [44,45].

### 2.4. Carbon Paste

**Figure 4:**
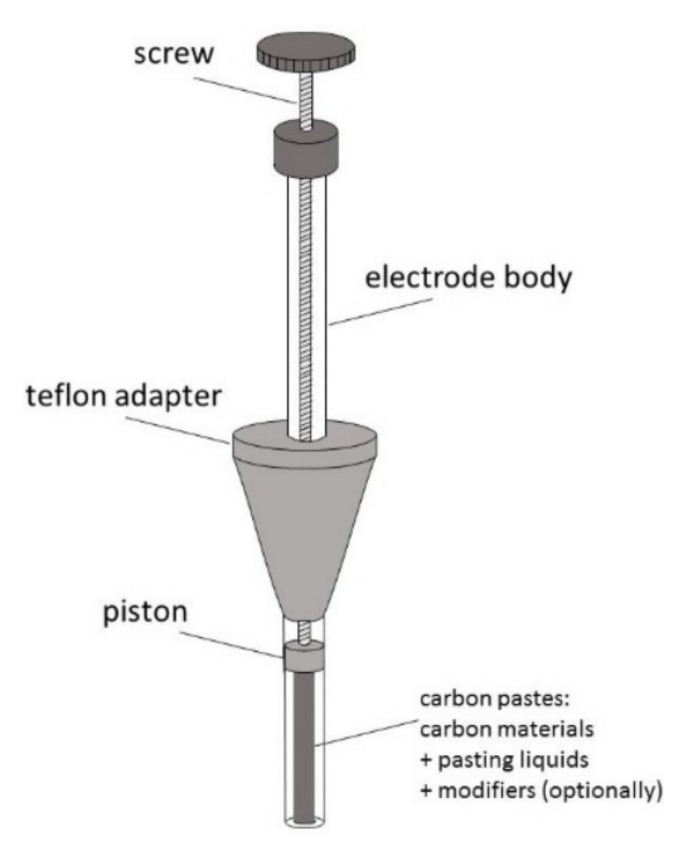
Schematic representation of carbon paste electrode.

As mentioned above, carbon paste (CP) was the first carbon material introduced to electroanalysis by Adams in 1958 [20]. Since then, CP has been widely used as an electrode material, and its basic composition has hardly changed [50]. In opposite to other carbon materials, this is a binary mixture, consisting of carbon particles mixed with various water-immiscible, of low volatility, high viscosity, and low solubility in the solvent of interest, chemically inert organic binders (pasting liquids) [50,51,52,53]. Most often, it is graphite powder (5–20 μm) dispersed in Nujol (mineral oil), less often—paraffin oil, hexadecane, or silicone oil and grease [18,50,51,52,53,54]. New CP composition may contain other than graphite forms of carbon such as glassy carbon (GCP), fullerenes (C60P), carbon nanotubes (CNP), graphene (GNP), and carbon fibers (CFP) [18,50,54,55,56,57]. These new forms of carbon are also added to CP as a modifier [18,52,54,56,57]. Typical carbon content in CP is about 70% by weight [18,56]. A homogenized carbon paste is placed into a tube made of glass, Teflon, or another isolating material, often equipped with a piston, to obtain a renewable disk electrode (CPE). The construction of the paste electrode is shown in Figure 4.

Renewing the surface is realized by replacing its outer layer and re-smoothing. The electrical contact provides a metal wire (e.g., Pt, Cu) immersed in the paste at the back of the disk. The electroactive surface area consists of tiny carbon micro-particles, which act as a microelectrode assembly with overlapping radial diffusion layers [18,50]. This provides the efficient mass transport of electroactive species to the surface of CPE. The exchange in electrons occurs at the contact of these particles with the electrolyte solution. Because of the hydrophobicity of pasting liquids and its ability to fill pores, an aqueous electrolyte cannot penetrate the paste. The electrode reactivity strongly depends on the paste composition. It was found that an increased content of the pasting liquid lowers the rate of electron transfer and background currents. A very important advantage of CP is its easy modification by adding a lot of different modifiers and thus constructing modified carbon pastes (MCPs) [18,50,51,52,53]. They can be introduced during the mixing of graphite and pasting liquid [51]. Both pasting liquids and modifiers are sometimes dissolved in a common solvent, which later evaporates [51,53,54]. The choice of modifiers depends on the electrode reaction mechanism studied. For instance, enzymes such as glucose oxidase, tyrosinase, laccase, or proteins are added to develop biosensors and insoluble solids (e.g., CaCO_3_, Cu, and other metal particles). After grinding and adding to the paste, they improve its selectivity or reactivity [18,50,51,52]. The amount of modifier in CP usually varies between 10 and 30% (*w/w*) [51]. It was found that CPEs can be an appropriate base for plating with metallic films (e.g., mercury, bismuth, gold) and thus offer properties similar to those of solid electrodes used in the construction of film electrodes. Such electrodes can be applied in electrochemical stripping methods [52,53]. The major advantages of CPEs are low cost, easy preparation and surface renewal, no risk of mechanical damage of the surface, very low background currents, a wide potential window, very low ohmic resistance, possibility of using various interactions (adsorption, extraction, ion-pairing, electrolysis, catalysis), and a variety of possible modifications [18,50,51,52,53]. Their main disadvantages are the tendency of the binder to dissolve in solutions containing an organic solvent, limited lifetime, the possibility of the interference of host material with redox reaction, and adsorption on carbon particles [51,52].

A group closely related to CPEs is Screen-Printed Carbon Electrodes (SPCEs). They are made of carbon inks printed and then hardened on an inert base (e.g., plastic or ceramic materials) [27,54,58,59]. The ink composition can be modified by the incorporation of different compounds, including carbon nanomaterials, metal oxides, noble metals nanoparticles, cobalt phthalocyanine, ionic liquids, which may enhance the selectivity and sensitivity of this sensor [27,54,58,59]. The reason for the versatile use of SPCEs is the possibility of mass production, and thus low costs, surface repeatability, planar configuration, and small dimensions. Particularly attractive is the possibility of their single-use (“one-shot”) and application as a sensor in a portable device, especially in environmental monitoring [50,58,59,60]. It should be emphasized that it is possible to construct a 3-electrode cell (working, counter, and reference electrodes) printed on the same strip. This miniature measurement cell allows reducing the analyzed sample to a small drop. The analytical application of these electrodes can also be extended by modification of their surface. SPEs can be modified with enzymes [18,58,59], MWCNTs [61,62], metal oxides, e.g., 3D CuO decorated amine-functionalized carbon nanotubes [63] or Fe_3_O_4_ [64], a base of plating with metallic films, e.g., antimony [58,59,60]. This in situ modified SPCE was successfully applied to the simultaneous determination of Pb(II), Cd(II), and Cu(II) ions, using anodic stripping voltammetry [60]. Interesting new SPCE surface modifiers are manganese ferrite (MnFe_2_O_4_), which is a good sensor applied to determination Hg(II) ions in real samples [65] and three-dimensional graphene oxide encapsulated cobalt oxide polyhedrons (3D GNO-Co_3_O_4_PHs) for voltammetric analysis of hydrogen peroxide [66].

### 2.5. Carbon Fibers

Carbon fibers (CF) have been used as an electrode material since the early 1980s [18,23,67]. They are made of polyacrylonitrile (PAN), polymer textile, or petroleum pitch, in a pyrolysis process similar to the one used for the manufacturing of GC [13,15,39,68,69,70]. The product of the pyrolysis process is then pulled out during curing. The catalytic chemical vapor deposition is also used [13]. The obtained CF has a cross-section of a “radial”, “onion”, or “random” type, with the end of fiber exhibiting a high fraction of the edge plane [15,18]. Therefore, this material is characterized by a well-ordered graphite-like structure and low porosity [13,18]. It can also be doped, e.g., with nitrogen or metals to obtain electrocatalytic properties [18,71]. CF diameters are from a few to about 60 µm. The fibers are embedded in glass with the use of epoxy, and then the carbon surface is exposed by cutting [18,67,68]. The carbon fibers electrodes (CFEs) are most often disk-shaped, less often of the cylinder. Due to the small diameters of CFEs (usually 5–15 µm), they are used as microelectrodes with characteristic radial diffusion of the analyte to their surface. The small surface area yields a low background current, a low ohmic drop error, *IR* (due to low faradaic currents) even at high scan rates, and thus they can be applied in highly resistive solutions [68,72]. Other advantages of microelectrodes are fast response time, fast kinetic, and small size, which means that electrochemical measurements need the use of a small number of samples. They are also successfully applied to investigations of processes in the living cells [18,23,67,68,70,73]. The improvement of CFE selectivity can be easily achieved by modifying their surface with various modifiers, e.g., with CNTs, enzymes [67,68], or plating of noble metals [18,67,68,70].

### 2.6. Carbon Nanomaterials

The rapid development of nanotechnology observed over the last few years has opened new opportunities in the construction of electrochemical sensors. Nanoelectroanalytical chemistry is a rapidly developing interdisciplinary field combining the advantages of electrochemistry with the unique properties of nanomaterials [23,54,74,75,76]. Electrochemical sensors can be applied to the detection of a wide range of analytes. They are characterized by low cost, high sensitivity and selectivity, simplicity, and fast response [54,76,77]. Among various electrode materials, carbon nanomaterials are used most often due to their unique physical and chemical properties such as easy preparation and modification, high conductivity, chemical stability, a wide potential range, and a low background current [54,76,77,78]. Apart from diamond and graphite, well-known allotropic varieties of carbon, new allotropes have been synthesized in the recent few decades—fullerenes, carbon nanotubes, and graphene (Figure 2). Due to their small sizes, they are included in a group called carbon nanomaterials. Each of them can be a component of various electrode materials with different electrochemical properties and can be applied in sensitive electroanalytical investigations of many inorganic and organic compounds, including biomolecules and drugs [21,25,54,74,75,76,78].

#### 2.6.1. Fullerenes

Buckminsterfullerene, the most well-known fullerene (C60), was discovered in 1985 by Kroto, Curl, and Smalley [78,79,80]. Following diamond and graphite, it is the third new allotrope of carbon. It was obtained by vaporizing graphite using laser irradiation. Buckminsterfullerene has also been found occasionally in nature [38]. The molecule consists of 60 carbon atoms (C_60_) arranged in a soccer ball structure (Figure 2) [79,81]. The surface of this cluster is formed by 20 hexagonal and 12 pentagonal carbon rings. Each carbon atom is covalently bound, and it is located at a corner where one five-membered and two six-membered rings come together [21,81]. This carbon allotrope is insoluble in polar solvents. However, it is partially soluble in benzene, toluene, and carbon disulfide [80]. The discovery of C60 opened a whole new area of carbon nanomaterials and carbon chemistry. The great importance of this discovery was awarded the 1996 Nobel Prize in Chemistry for Kroto, Curl, and Smalley [23,77,80]. Since then, other fullerenes with different sizes of carbon cages have been synthesized, e.g., C_20_ C_70_, C_78_, C_84_, C_140_, C_260_ [21,23,38,77,78]. All fullerenes are based on sp^2^ hybridized carbon, such as glassy carbon and graphite, but differ in their spatial structure. A large specific surface area of fullerenes, a low degree of aggregation, mechanical resistance, ability to become a superconductor when combined with alkali metals, chemical stability, electrocatalytic properties, and a wide positive potential range makes them useful as modifiers of electrode surfaces, especially of the pastes in carbon paste electrodes [19,21,22,77]. Due to the limited solubility of fullerenes, their functionalization aimed at giving them hydrophilic or amphiphilic properties has become an important problem [81]. Fullerenol with -OH groups is an example of a water-soluble compound, and metallofullerenes are examples of hydrophobic derivatives of C_60_ with catalytic properties in many organic reactions [80]. Fullerene derivatives are often semiconducting materials in which electrons are charge carriers, and thus they can be applied in the construction of sensors. However, due to high synthesis costs and low yields of the methods currently available for the production of fullerenes, their widespread use is limited [21].

#### 2.6.2. Carbon Nanotubes

Carbon nanotubes (CNTs) were discovered thirty years ago (1991) by Sumio Iijima [82]. Their name originates from the shape of a tube with a nanometer diameter. CNTs were first obtained using an arc-discharge evaporation method, similar to the one used for the preparation of fullerenes. Currently, three main methods are used for this purpose: arc-discharge, laser ablation, and chemical vapor deposition (CVD) on metal particles, often Fe or Ni [18,21,76,81,82,83]. CNTs are well-ordered structures consisting of single layers of graphite (graphene) rolled into the form of cylinders (tubes) with an sp^2^-hybridized carbon atom [21,83,84]. They can be divided into two categories: single-walled carbon nanotubes (SWCNTs), which consist of a single graphene sheet rolled into a tube, and multi-walled carbon nanotubes (MWCNTs) containing several concentric tubes with a common axis (Figure 2) [21,27,76,77,78,83,84]. Depending on the type of nanotubes, their diameters range from 0.4–2 nm to 2–100 nm for SWCNTs and MWCNTs, respectively, whereas the lengths range from one hundred nanometers to several millimeters [77,84]. The space between tubes in MWCNTs (approx. 0.42 nm) is comparable to the interlayer spacing in graphite [56,76]. The unique structure of CNTs results in their unusual properties, including small size, large specific surface area, high ratio of length to diameter (aspect ratio), flexibility, high mechanical and tensile strength, high heat resistance, excellent high thermal conductivity, high chemical stability, extremely low electrical resistivity, and the ability to entrap atoms of other elements within their molecular structure [21,23,27,76,77,83,84]. The last property causes CNTs to be easily modified by various nanomaterials, e.g., nanoparticles, by attachment of molecules, such as enzymes, aptamers, or redox-active compounds, or by introducing a lot of different surface functional groups (e.g., -COOH, -OH, or -C=O) [22,23,54,76,77,83,85,86]. The extraordinary properties of CNTs make them the most widely applied electrode material in electroanalysis [18,26]. They are used both in bare and functionalized forms to modify the surface of solid electrodes. Most frequently, CNTs are deposited onto a conducting surface, such as glassy carbon or graphite, by evaporating their suspension in bromoform, ethanol, or in N,N-dimethylformamide [26,83,85]. The increased electroactive surface area of CNT-based electroanalytical sensors and often their electrocatalytic activity make them exhibit good conductivity and high chemical stability, fast and reproducible responses, the ability to resolve the overlapped responses of analytes, higher sensitivity, lower limits of detection, and faster electron-transfer kinetics, compared with traditional electrodes such as GCE or CPE [23,54,83].

#### 2.6.3. Graphene

Graphene (GN) is a thin sheet carbon allotrope that was received by Novoselov and Geim in 2004 [87,88,89]. They were awarded the 2010 Nobel Prize in Physics for their seminal work on this new nanocarbon material [23,75,90]. GN is the name of a single sheet of carbon atoms tightly packed into a two-dimensional (2D) hexagonal honeycomb lattice with a bond distance of 0.142 nm [21,23,81,88,90,91]. This nanomaterial is a basic building element for other carbon allotropes (Figure 2). It can be wrapped in 0D fullerenes, rolled into 1D nanotubes, or stacked into 3D graphite [21,22,27,81,85,88,89,90,91]. It was initially produced by micromechanical cleavage of bulk graphite [87,88]. However, the yield of this method is very low, and the process is difficult to control [19,74]. Nowadays, various methods of graphene obtaining are applied, including direct synthesis from graphite or its derivatives, chemical vapor deposition on metal substrates, thermal decomposition of SiC, substrate-free gas-phase synthesis, chemical reduction in graphene oxide, and even electrochemical synthesis [19,74,76,81,84,88,89,90,91,92,93]. However, it is practically difficult to obtain a single layer of graphene in a controlled manner [81,84,88,91]. For this reason, carbon nanomaterial can be considered as graphene when it poses single-, double-, or several (3 to <10) layers of sp^2^-hybridized carbon atoms arranged in six-membered rings. This makes it possible to distinguish between three different types of graphene [74,77,88,91]. Thicker structures should be treated as thin films of graphite [88]. The unique 2D structure of graphene results in its extraordinary properties, including mechanical, chemical, optical, thermal, and electrical ones. GN is described as the thinnest, most flexible, and strongest material to be known [81,90]. It is mechanically stable, fracture strong, but harder than diamond [19,23,75]. Graphene has a small size and extremally large specific surface area (2630 m^2^ g^−1^), which is twice as large as that of CNTs (1315 m^2^ g^−1^) [74,76,90,91]. GN is chemically stable and poses high optical transmittance (up to 97.7% [21]) and high thermal conductivity. Its semi-metallic band structure makes it conduct electricity faster at room temperature than any other material (calculated conductivity 64 mS cm^−1^ is approximately 60-fold greater than that of SWCNTs) [19,23,75,91,92]. Due to the existence of different forms of graphene, these unique electrical properties are not fully explained [84,93]. However, significant evidence exists that GN may exhibit very good electrochemical performance compared with other electrodes, such as GC or even CNTs [84,92,93]. In the opinion of many, the emergence of graphene means progress in nanocarbon electrochemistry and electroanalysis [23]. A large 2D aromatic surface of GN and related materials, e.g., graphene oxide (GNO) or chemically reduced graphene oxide (CR-GNO), is a promising base for the synthesis and design of multicomponent electrode materials [19,54,85,91,93]. Moreover, the incorporation of both non-metallic heteroatoms (B, S, N, and P) and transition metals (Pd, Pt, Rh, Ag, Ni, Co, Mn, and Fe) as dopants enhances the utility of GN as electrode material [23,74,85,91]. The presence of oxygen-containing groups at its edges or surfaces additionally facilitates the attachment of different specific groups [89,91]. Thus, several organic compounds are introduced to GN, GNO, and CR-GNO, such as DNA, aptamers, proteins, peptides, cellulose, hemoglobin, cytochrome B, enzymes to construct high sensitivity electrochemical biosensors [17,38,45,47,48,57,58,61,62,63,64,65]. It was found that CR-GNO has more advantages as an electrode material than GNO does [19,92]. The electrochemical sensors based on GN are made as a modification of solid electrodes, mainly of GC [19,25,54,74]. GN effectively promotes the electron transfer between electrode and analyte and thus enhances their analytical signals [92,93]. Graphene-based electrodes show higher stability, better conductivity, higher electrocatalytic activity, as well as greater currents at lower potentials in comparison with CNTs-based ones.

#### 2.6.4. Other Carbon Nanomaterials

Carbon nanoparticles (CNPs) are newly emerged carbon nanomaterials, which are increasingly a component of novel electrochemical sensors. The best-known carbon nanoparticles are undoped/doped carbon nanodiamonds (CNDs), carbon nanohorns (CNHs), carbon quantum dots (CQDs), graphene quantum dots (GNQDs), carbon nanofibers (CNFs), and carbon black (CB) [21,23,54,74,86,94]. They are known for their small size, low cost, easy synthesis, good electrical conductivity, chemical stability, and biocompatibility. Moreover, CNPs have a high surface area with a large adsorption capacity; they are easy to embed chemical and biological molecules and functionalize. Thus, they are useful for the production of nanocomposite electrode materials with electrocatalytic properties [23]. One of the newest carbon nanomaterials is graphitic carbon nitride (G-C_3_N_4_)—A two-dimensional layered structure consisting of C and N connected thorough tris-triazine patterns which, when composited with zirconium phosphate (α-ZrP) [36] or with MWCNTs [37], shows unique electrochemical features.

## 3. Electroanalysis of Preservatives on Carbon Materials

As described above, preservatives are substances that protect a product against the growth of microorganisms such as bacteria, fungi, and molds [1,2]. Their task is to maintain appropriate quality and microbiological purity and thus prolong the shelf-life of a product. Preservatives are characterized by a diverse chemical structure. Most often, they belong to the group of quaternary ammonium bases, organic mercury compounds, biguanides, alcohols, phenols, aldehydes, and organic acids [95].

The literature data show that 432 different preservatives were used in 2010. The most numerous group is isothiazolines and other compounds containing nitrogen. Phenols and benzoic acid derivatives are the second most frequently used. The proportion of inorganic preservatives is the lowest and does not exceed 1% [95]. The controversial influence of preservatives on the human body, and their widespread use, make it necessary to determine properties, including electrochemical ones, and to develop effective methods of their determination in everyday products. Besides, dominant chromatography and voltammetric techniques are increasingly used for this purpose.

**Figure 5:**
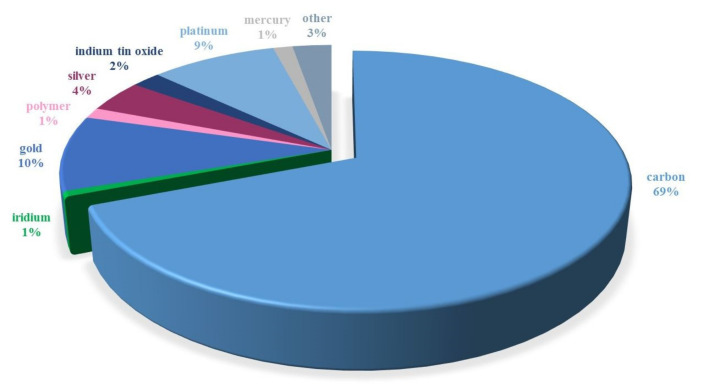
Frequency of use of electrode materials in the electroanalysis of preservatives from 2000 to 2021 (data obtained from Scopus).

The voltammetric determination of preservatives is based on their anodic oxidation and/or cathodic reduction. The measurement process takes place on a polarizable working electrode made of different materials. Over the last 21 years, carbon in various forms (Figure 5) has been the most popular electrode material used for the voltammetric determination of preservatives. It was followed, in terms of frequency of use, by gold [96,97,98,99,100] and platinum [101,102,103,104,105,106,107]. The remaining electrode materials were rarely used. One such material is mercury. Due to its toxicity and the narrow range of anodic potentials, it is less and less often used in the analysis of preservatives [108,109,110]. Since the share of carbon materials used in the electroanalysis of preservatives is the highest and reaches about 69%, the literature on this subject was reviewed.

Carbon sensors are most often used for the voltammetric analysis of parabens (MP, EP, PrP, BP) [28,29,111,112,113,114,115,116,117,118,119,120,121,122,123,124,125,126,127,128,129,130,131,132,133], synthetic antioxidants (BHT, BHA, TBHQ, PrG) [30,32,61,134,135,136,137,138,139,140,141,142,143,144,145,146,147,148], benzoic acid (BA) [149,150,151,152,153], and other preservatives. The most commonly used measurement technique is differential pulse (DPV) or square-wave (SWV) voltammetry. Less common is linear sweep (LSV) or cyclic (CV) voltammetry. In most cases, the environment used for the analysis of preservatives is aqueous buffer solutions, although mixed non-aqueous solvents also appear [118,136]. Table 1 presents the overview of voltammetric methods for the determination of main preservatives in real samples using different electrolytes and various carbon materials, as well as the crucial analytical parameters: limits of determination (*LOD*) and linearity ranges (*LR*).

Among the electrode materials used for the voltammetric methods analysis of preservatives, the dominant one is glassy carbon (GC), both bare [122,129,131,144,145,154] and with various modifications such as multi-walled carbon nanotubes (MWCNTs) [113,128,132,139,155,156,157,158], single-walled carbon nanotubes (SWCNTs), fullerenes [114], graphene composites [111,115,143,159,160], α-zirconium phosphate composited with graphitic carbon nitride (α-ZrP@G-C_3_N_4_) [36], nafion (NAF) [128,132,155], nanoparticles of metals [112,123,127], metal oxides [29,30,31,32,161], and organic compounds [121,162]. There are also several papers presenting the use of nanomaterials, such as graphene quantum dots (GNQDs) [94] or CNFs [112,127], as glassy carbon electrode surface modifiers. The analytical parameters (Table 1) obtained on the presented electrodes indicate that the modification of glassy carbon surface lowers the detection limits in relation to bare GC. For example, the use of MWCNTs/NAF/GCE electrodes allows the determination of 8-hydroxyquinoline (8-HQ) at the concentration level of 9 nM [155]. GNQD modified glassy carbon electrode has been applied to the determination of thiomersal in influenza vaccine using square-wave voltammetry [94]. This sensitive indirect method, based on Hg/Hg^2+^ redox peak, is characterized by a low limit of detection (0.9 μM) and a linearity range from 3.0 to 32 μM (Table 1). It should be noted that determination of the same analyte on a renewable mercury film electrode, Hg(Ag)FE using adsorptive stripping voltammetry [110], allowed to achieve a much lower detection limit (0.9 nM) compared to those obtained on modified GCE [94] and CPE [163] (Table 1). This means that, despite its toxicity, mercury is still a very good electrode material, also for the determination of preservatives based on their cathodic reduction processes. Graphene quantum dots (GNQDs), together with silver nanoparticles, carbon nitride nanotubes, and the ionic liquid was also used to modification GCE and construct a new sensor for the determination of triclosan in wastewater samples [164]. This modification allows achieving *LOD* on the extremely low level of 2.0 × 10^−3^ nM. The comparison of the results of MP determination obtained on a bare and with different modifiers GCE surface [111,113,123] clearly indicates that surface modification enables to achieve better analytical parameters (Table 1). The same conclusions can be drawn in the case of other analytes presented in Table 1, e.g., EP. Modification of GCE with functionalized fullerene nanorods [114], the composite of carbon nanofibers (CNFs), and tri-metallic nanoparticles (Au-Ni-Co) [112] make it possible to obtain *LOD* of 3.8 nM and 0.35 nM, respectively. The use of carbon nanofibers (CNFs), nanotubes (CNTs), and nanoparticles of various metals for the modification of glassy carbon enable a specific reduction in *LOD* value to the level of nM in the analysis of parabens [112,123,127]. This method of electrode modification also allows obtaining wide ranges of linearity.

The second frequently used material is carbon paste (CP). It is mainly based on graphite [116,119,120,124,135,163,165,166] but there are also studies using pastes made of glassy carbon (GCP) [167], graphene (GNP) [168], and multi-walled carbon nanotube (MWCNTP) [169]. The modifiers of carbon paste are usually MWCNTs [124,135,165], nanoparticles of inorganic compounds (Zn(OH)_2_ [116], ZnO [119], LaO_x_ [163]) and various polymers [119,166], or molecularly imprinted polymer (MIP) [120]. Using MIPs as a modifier of carbon paste makes it possible to obtain a low *LOD* value of propylparaben (0.32 nM [120]).

**Table 1:** Overview of voltammetric methods of preservatives determination on bare and modified carbon electrodes.

Electrode		Analyte	Real Sample	Electrolyte	Voltammetric Technique	*LR* μM	*LOD* μM	Ref.
BASE	MODIFIER							
GC	BARE	BHA BHT TBHQ PrG	Food	HClO_4_/MetOH	LSV	2.8–83.2 2.3–36.3 6.0–90.2 4.7–70.7	1.05 0.68 0.44 2.54	[144]
		BHA BHT TBHQ	Food	HCl/H_2_O	SWV	11.1–554.8 36.3–90.8 6.0–481.3	nd	[145]
		BHA BHT TBHQ		BRB pH 2.0		11.1–443.8 18.2–136.1 24.1–481.3		
		MP	Pharmaceuticals Cosmetics	HClO_4_/H_2_O	SWV	10–202	3.28	[129]
		MP EP PrP BP	Food	BRB pH 4.5	SWV	0.78–4.48	0.29 0.38 0.36 0.39	[122]
		BP	Water	K_4_P_2_O_7_, CTAC	DPV	0.1–1000	0.1	[131]
		BAC	Pharmaceutical	TBAH/AN	SWV	10–200	1.7	[154]
GC	MWCNTs	CAR	Food	PBS pH 6.5	DPV	0.1–150	0.075	[157]
	MWCNTs/NAF	8-HQ	Cosmetics	Ac-B pH 3.6	DPV	0.02–10	9 × 10^–3^	[155]
		MP	Standard solution	PBS pH 6.5	LSV	3–100	1.0	[128]
		BP	Water	PBS pH 7.0	AdSV	10–100	0.2	[132]
	MWCNTs-LB	MP	Cosmetics	PBS pH 3.0	LSV	1–80	0.4	[113]
	3D GN-MWCNTs	Natamycin	Food	H_2_SO_4_/H_2_O	LSASV	0.05–2.5	0.01	[156]
	poly(carminic acid)/MWCNTs	BHA TBHQ	Oil	BRB pH 2.0	DPV	0.25–75 0.50–75	0.23 0.36	[139]
	PTZ-IL/MWCNTs	Sulfite	Food	NH_4_Cl/H_2_O	AMP	30–1177	9.3	[158]
	GN-SWCNTs/MIPs	PrG	Food	PBS pH 6.0, KCl	DPV	0.08–2600	0.05	[170]
	SWCNTs/poly(L-serine)	Natamycin	Food	H_2_SO_4_ pH 1.0	LSV	0.06–6.0	0.04	[171]
	Pt-NP@SWCNTs	MP	Standard solution	PBS pH 7.0	DPV	5.0 × 10^–3^–0.03	5.0 × 10^–3^	[123]
	(Co-Ni-Pd)NPs-CNFs	MP	Pharmaceuticals Cosmetic Urine	PBS pH 7.0	SWV	3 × 10^–3^–0.3	1.2 × 10^–3^	[127]
	(Au-Ni-Co)NPs-CNFs	EP	Cosmetics Pharmaceuticals	PBS pH 7.0	SWV	1.0 × 10^–3^–0.1	3.5 × 10^–4^	[112]
	AuNPs	BHA BHT TBHQ	Food	BRB pH 2.0	LSV	0.55–8.32 0.91–9.98 1.2–16.8	0.22 0.36 0.48	[142]
	Pt-Pd NPs/CS/N-GN	Sulfite	Pharmaceutical	PBS pH 4.0	DPV	8–600	5.5	[172]
	GN-CS/AuNPs	Sulphite Nitrite	Water	PBS pH 7.5	AMP	5–410 1–380	1 0.25	[159]
	R-GNO/ZnO	Formaldehyde	Urine	PBS pH 7.4 hexamine	CV	nd	0.023	[31]
	R-GNO-CS/AuNPs	MP	Standard solution	PBS pH 8.0	SWV	0.03–1.3	0.014	[115]
	R-GNO/RuNPs	MP	Cosmetics	PBS pH 7.0	DPV	0.50–3.00	0.24	[111]
	ERC60NRs-NH-Ph	EP	Cosmetics	PBS pH 7.0	SWV	0.01–0.52	3.8 × 10^–3^	[114]
	IrOxNPs	BHA	Standard solution	PBS pH 2.0	CA	1–280	0.6	[30]
	CuO/Cu_2_O-CPL6	PrP	Standard solution	PBS pH 3.0	DPV	1–35.0	0.46	[29]
	CuV_2_O_6_ NBes	BA	Standard solution	KCl/H_2_O	CV	1–2000	0.61	[150]
	PTh/CuBi_2_O_4_ NSNCs	BA	Water	KCl/H_2_O	CV	1–2000	0.56	[151]
	CuGeO_3_ NWs	BA	Standard solution	KCl/H_2_O	CV	1–2000	0.91	[149]
	PANI/CuGeO_3_ NWs	BA	Standard solution	KCl/H_2_O	CV	1–2000	0.96	[153]
	In_2_O_3_ NBrs	BP	Cosmetics	PBS pH 7.0	SWV	0.14–2.4	0.08	[28]
	MnO_2_/R-GNO	TBHQ	Oil	PBS pH 7.0	DPV	1.0–50.0; 100.0–300.0	0.8	[32]
	MoS_2_/NAF	Sulfite	Water	Ac-B pH 3.6	DPV	5–500	3.3	[173]
	LaFeO_3_/GN	Sulfite	Food	PBS pH 7.0	DPV	1–200	0.21	[174]
	GN/Ch	TBHQ BHA	Food	PBS pH 3.0	DPV	0.40–120 0.60–200	0.14 0.19	[143]
	PPy	MP	Cosmetics	BRB pH 5.0/AN	DPV	10–5000	8.0	[121]
	PPy-CS	Sulfite	Food	PBS pH 8.5	DPV	50–1100	0.21	[175]
	Fe_3_O_4_@Au-PPy/GO	TCS	Cosmetics Urine	PBS pH 9.0	DPV	0.01–1.0	2.5 × 10^−3^	[161]
	ANSA	8-HQ	Cosmetics	BRB pH 2.0	SWV	0.5–425	0.16	[162]
	MIPs	MP, EP PrP BP	Cosmetics	PBS pH 6.5	SWV	20–100 5–100 5–80	0.4 0.2 0.2	[130]
	MIPs/PtAu-GN-MWCNTs	PrG	Oil	PBS pH 6.5 K_3_[Fe(CN)_6_], KCl	CA	0.07–10	0.025	[176]
	PDDA-GN/PdNPs	TCS	Standard solution	PBS pH 7.0	DPV	9 × 10^−3^–20	3.5 × 10^−3^	[160]
	GNQDs	Thiomersal	Influenza vaccines	BRB pH 4, KCl	SWV	3.0–32	0.9	[94]
	AgNPs/C_3_N_4_NTs@GNQDs/ILs	TCS	Wastewater	BRB pH 9.0	DPV	1.0 × 10^−5^–0.01	2.0 × 10^−6^	[164]
	α-ZrP@G-C_3_N_4_	Nitrite	Food	PBS pH 7.0	DPV AMP	0.01–173 0.002–436	5 × 10^–3^ 7 × 10^–4^	[36]
	LuHCF/poly(taurine)	Sulfite	Food	KCl/H_2_O	DPV	nd	1.33	[177]
	Au_3_Pd_4_	TBHQ Nitrite	Oil	PBS pH 6.5	DPV	2–4200 2–200	0.67 nd	[141]
	Au_3_Pt_3_	TBHQ	Oil	PBS pH 7.0	DPV	0.35–625	0.075	
	MTF/sulfite oxidase	Sulfite	Food	PBS pH 7.0	DPV	200–2800	nd	[178]
BDD	BARE	Natamycin	Pharmaceuticals	H_2_SO_4_	SWV	0.89–8.26	0.20	[179]
				H_2_SO_4_, SDS		0.098–1.16	0.03	
		BHA BHT	Food	KNO_3_/H_2_O/EtOH	SWV	0.60–10 0.60–10	0.14 0.25	[146]
		BAC	Pharmaceuticals	TBAH/AN	SWV	10–200	1.7	[154]
		MP EP PrP	Aqueous matrix	Na_2_SO_4_ pH 7.0 EtOH/water	CV	2–104 20–180 20–140	1.50 1.97 3.60	[117]
		MP EP PrP			CA	10–80 2–112 10–80	0.70 1.03 0.97	
MWCNTs	POC	BHA	Food	PBS pH 6.0	DPV	0.33–110	0.11	[138]
CP	MWCNTs	sulphite SO_2_	Food	BRB pH 1.0	SWV	25–500	16	[165]
	MWCNTs/Hb	MP	Urine Human serum	PBS pH 7.0	DPV	0.1–13	0.025	[124]
	MWCNTs-NAF-SEPperox	TBHQ	Food	PBS pH 7.0	SWV	9.93–59.08	2.47	[135]
	MIPs	PrP	Cosmetics	PBS pH 7.0	DPV	1 × 10^–3^–0.1	3.2 × 10^–4^	[120]
	PVI	Nitrite	Food	PBS pH 4.0	DPV	0.5–100	0.09	[166]
	FeNi_3_/R-GNO/HMPF_6_	TBHQ	Food	H_2_O/EtOH pH 7.0	SWV	0.05–900	0.01	[140]
	NiTiO_3_	OHB PHB	Cosmetics	BRB pH 5.0	DPV	10–1000 10–1000	0.39 0.10	[180]
	LaOx	Thiomersal	Vaccines Pharmaceuticals	PBS pH 3.0	SWSV	1.0–10.0	0.09	[163]
	PANI/ZnO	PrP	Standard solution	BRB pH 5.0	DPV	1.0–100.0	0.13	[119]
	Zn(OH)_2_-NPs	MP EP PrP BP	Standard solution	PBS pH 7.0	DPV	4–1255 100–1500 40–1050 11–230	3.21 4.01 11.30 3.12	[116]
		MP EP PrP BP			CV	12–360 100–300 14–430 75–160	5.00 34.04 11.35 9.90	
GCP	BARE	8-HQ	Standard solution	BRB pH 9.0/MetOH	DPV	0.1–100	0.052	[167]
GNP	BF/IL	Sulphite	Water	PBS pH 8.0	SWV	0.05–250	0.02	[168]
MWCNTP	SLS	8-HQ	Standard solution	PBS pH 7.0	CV	20–1000	0.11	[169]
SPC	BARE	SA	Food	BRB pH 2.0, NaCl	DPV	1–200	1.6	[181]
SPC	MWCNTs	TBHQ BHA	Biodiesel	BRB pH 2.0/MetOH CTAB	LSV	0.50–10 0.50–10	0.34 0.18	[61]
		KA	Food	BRB pH 2.2	DPV	20–5000	16	[62]
	Pt-CdS/MWCNTs	Natamycin	Food	H_2_SO_4_ pH 1.0	DPV	0.2–70.0	0.12	[137]
	CuO NFs/NH_2_-MWCNTs	TBHQ	Oil	PBS pH 6.0	DPV	0.01–147.6	3 × 10^–3^	[63]
	Fe_3_O_4_	Nitrite	Standard solution	BRB pH 8.0	SWV	nd	0.013	[64]
	CoSe_2_@R-GNO	PrG	Food	PBS pH 7.0	DPV	0.075–460.2	0.016	[182]
	CNC-R-GNO	MP	Cosmetics	PBS pH 7.0	DPV	200–900	100	[133]
	3D GNO-Co_3_O_4_PHs	H_2_O_2_	Disinfectant cleaning solutions	PBS pH 7.0	AMP	0.05–400; 450–1250	0.015	[66]
CF	BARE	BHA BHT	Pharmaceuticals	AcH-AN, NaClO_4_	DPV	0.17–3299 0.82–4181	0.06 0.27	[136]
		MP PrP	Pharmaceuticals	AcH-AN, AcNa	DPV	5.85–267.7 6.66–203.2	0.52 0.55	[118]
		SA	Pharmaceutical	PBS pH 7.4	DPV	2.0–3000	1.68	[183]
		MCI MIT	Cosmetics	LiClO_4_/H_2_O	DPV	26.7–1538 17.4–2259	nd	[184]
G	NAPCF	BHA	Food	NaNO_3_, PBS pH 7.0	CV CA	0.62–219 0.58–5.03	0.25	[148]
EPPG	SWCNT-Co	Nitrite	Standard solution	PBS pH 3.0 PBS pH 7.4	AdSV	Nd	11.6 8.4	[185]
PG	*p*-Phe-MP	MP	Pharmaceuticals Cosmetics	BRB pH 2.0	DPV	10–5000	10	[125]
	*o*PPy-*β*-CD-PMo_12_	PrP	Cosmetics	BRB pH 6.0	DPV	0.2–100	0.04	[126]
GWc	CoHCF	BHA	Food	NaCl/H_2_O	CA	0.79–190	0.19	[134]
	Teflon–tyrosinase	BA	Food	PBS pH 7.4, AOT	AMP	1.0–40	0.90	[152]
MCc	Cu_3_(PO_4_)_2_-Poly	BHA BHT	Food	KNO_3_ pH 6.7/EtOH	SWV	0.34–41	0.072 0.093	[147]

**Ac-B**—acetate buffer; **AcH**—acetic acid; **AcNa**—sodium acetate; **AdSV**—adsorptive stripping voltammetry; **AgNPs/C_3_N_4_NTs@GNQDs/ILs**—silver nanoparticles/carbon nitride nanotubes@graphene quantum dots/5-nitro-2-(3-hydroxy-4-methoxybenzylidenamino)-thiazole **AMP**—amperometry; **AN**—acetonitrile, **ANSA**—1-amino-2-naphthol-4-sulfonic acid; **AOT**—dioctyl sulfosuccinate; **Au_3_Pd_4_**—dumbbell-style AuPd; **Au_3_Pt_3_**—dendrite-like AuPt; **BA**—Benzoic acid; **BAC**—Benzalkonium chloride; **BDD**—Boron-Doped Diamond; **BF/IL**—benzoylferrocene/ionic liquid; **BHA**—butylated hydroxyanisole; **BHT**—butylated hydroxytoluene; **BP**—Butylparaben; **BRB**—Britton–Robinson buffer; **CA**—chroanamperometry; **CAR**—carvacrol; **CF**—Carbon fiber; **Ch**—choline; **CNC**—cellulose nanocrystal; **CNFs**—carbon nanofibers; **CoHCF**—cobalt hexacyanoferrate; **CoSe_2_@R-GNO**—reduced graphene sheets with cobalt diselenide nanoparticles; **CP**—carbon paste; **CS**—chitosan; **CTAB**—cetyltrimethylammonium bromide; **CTAC**—cetyltrimethylammonium chloride; **CuO NFs/NH_2_-MWCNTs**—three-dimensional CuO nanoflowers with functionalized multi-walled carbon nanotubes; **CuO/Cu_2_O-CPL6**—Printex L6 (CPL6) carbon black modified with copper oxides nanoparticles; **CV**—cyclic voltammetry; **3D GN**—three-dimensional graphene; **3D GNO-Co_3_O_4_PHs**—three-dimensional graphene oxide encapsulated cobalt oxide polyhedrons; **DPV**—differential pulse voltammetry; **EP**—Ethylparaben; **EPPG**—edge plane pyrolytic graphite; **ERC60NRs–NH–Ph**—electrochemically reduced fullerene nanorod; **FeNi_3_/R-GNO/HMPF_6_**—reduced graphene oxide/FeNi_3_-ionic liquid (n-hexyl-3-methylimidazolium hexafluoro phosphate); **Fe_3_O_4_@Au-PPy/GO**—Fe_3_O_4_@Au nanostructure decorated GO with polypyrrole (PPy), **G**—graphite; **GC**—glassy carbon; **GCP**—Glassy Carbon Paste; **GN**—graphene; **GNP**—graphene nano-sheets paste; **GNQDs**—graphene quantum dots; **GWc**—graphite paraffin wax composite; **Hb**—haemoglobin; **8-HQ**—8-Hydroxyquinoline; **KA**—kojic acid; **LaFeO_3_/GN**—lanthanides orthoferrites with graphene composite; **LaO_x_**—Lanthanum oxides; **LB**—Langmuir-Blodgett film; **LSASV**—linear sweep adsorptive stripping voltammetry; **LSV**—linear sweep voltammetry; **LuHCF/poly(taurine)**—Lutetium(III) hexacyanoferrate microparticles electrodeposited on poly(taurine); **MCc**—modified carbon composite; **MCI**—methylchloroisothiazolinone; **MIPs**—molecularly imprinted polymers film; **MIT**—methylisothiazolinone; **MnO_2_/R-GNO**—MnO_2_ electrodeposited onto the electrochemically reduced graphene oxide; **MoS_2_/NAF**—molybdenum disulfide and Nafion; **MP**—Methylparaben; **MTF/sulfite oxidase** –sulfite oxidase immobilised on mercury thin film; **MWCNTP**—Multi-walled carbon nanotube paste; **MWCNTs**—multi-walled carbon nanotubes; **NAF**—Nafion; **NAPCF**—nickel aquapentacyanoferrate; **NBes**—nanobelts; **NBrs**—nanobricks; **NPs**—nanoparticles; **NSNCs**—nanosheet nanocomposites; **NWs**—nonowires; **OHB**—*o*-hydroxybenzoic acid; ***o*PPy-β-CD-PMo_12_**—polypyrrole (PPy) grafted by the organic–inorganic β-Cyclodextrin-phosphomolybdate; ***p*-Phe-MP**—*p*-phenylenediamine conducting polymer imprinted with methyl paraben; **PANI**—polyaniline; **PBS**—phosphate buffer; **PDDA-GN/PdNPs**—poly (diallyldimethylammonium chloride) functionalized graphene/palladium nanoparticles; **PG**—pencil graphite; **PHB**—*p*-hydroxybenzoic acid; **POC**—poly *o*-cresophthalein complexone; **PPy**—polypyrrole; **PPy-CS**—polypyrrole-chitosan thin film; **PrG**—propyl gallate; **PrP**—Propylparaben; **PTh**—Polythiophene, **Pt-Pd NPs/CS/N-GN**—Pt-Pd bimetallic nanoparticles on chitosan/nitrogen doped graphene; **PTZ-IL/MWCNTs**—phenothiazine imidazoliumionic liquid with hexafluorophosphate counter anion immobilized onto multiwalled carbon nanotubes; **PVI**—polyvinylimidazole; **R-GNO**—reduced graphene oxide; **R-GNO/ZnO**—micro-dumbbell shaped ZnO rods on reduced graphene oxide; **SA**—Salicylic acid; **SEPperox**—peroxidase immobilised on sepiolite; **SDS**—sodium dodecyl sulfate; **SLS**—Sodium lauryl sulfate; **SP**—screen-printed; **SPC**—screen-printed carbon; **SWCNTs**—single-walled carbon nanotubes; **SWSV**—square wave stripping voltammetry; **SWV**—Square wave voltammetry; **TBAH**—tetrabutylammonium hexafluorophosphate; **TBHQ**—butylated hydroquinone; **TCS**—triclosan; **α-ZrP@G-C_3_N_4_**– α-zirconium phosphate composited with graphitic carbon nitride.

Screen-printed electrodes (SPEs) are also used, most often modified with MWCNTs [61,62] or their combinations Pt-doped CdS nanoparticles [137], or reduced graphene decorated with cobalt diselenide (CoSe2@R-GNO) [182] or cellulose nanocrystal (CNC-R-GNO) [133].

Boron-doped diamond electrode (BDDE) is increasingly popular as the material of the working electrode used in the electroanalysis of preservatives [117,146,179]. The use of SWV on BDDE allows the analysis of natamycin and synthetic antioxidants with low *LOD* values (Table 1). An additional factor enabling *LOD* of natamycin reduction may be the use of the addition of sodium dodecyl sulfate (SDS) as a surfactant [179].

In addition to the above-mentioned electrode materials, composite electrodes based on graphite paraffin wax composite (GWc) [134,152], or modified carbon composite (MCc) [147], are also used in the voltammetric analysis of preservatives. Carbon composite modified with copper(II) phosphate immobilized in a polyester resin (Cu_3_(PO_4_)_2_-Poly), used as the working electrode for the analysis of BHA and BHT, allowed obtaining very low *LOD* values: 0.072 and 0.093 μM, respectively.

As can be seen, the voltammetric determination of preservatives is more often carried out with modified rather than unmodified electrodes. This tendency will most probably continue in the coming years.

## 4. Conclusions

The usefulness of modern electroanalysis, especially voltammetric techniques, for solving real-life analytical problems is closely related to the appropriate material of working electrodes. Their use should ensure obtaining the best electroanalytical parameters with simultaneous ease of use and safety for humans and the environment. Carbon in various forms, especially with modifications to its surface, undoubtedly follows this trend. The growing popularity of carbon materials is related not only to chemical inactivity, excellent electrochemical and analytical properties but also to their low cost and being eco-friendly.

These advantages are the reason for an ever-increasing interest in the use of carbon materials in electroanalysis. Their usefulness was demonstrated in examples of preservatives. The carbon-based sensors ensure appropriate selectivity, high sensitivity, low limits of detection and quantification, as well as wide linearity ranges of voltammetric analysis of these popular compounds. Their application in this domain constantly increases. Such tendency is likely to continue in the following years. The present review should help anyone involved in voltammetric techniques with the analysis of preservatives and other biochemically active compounds in real samples.

## Data Availability

Data available in cited publications.
